# *Leukocyte Tyrosine Kinase* (*Ltk*) Is the Mendelian Determinant of the Axolotl Melanoid Color Variant

**DOI:** 10.3390/genes14040904

**Published:** 2023-04-13

**Authors:** Mirindi Kabangu, Raissa Cecil, Lloyd Strohl, Nataliya Timoshevskaya, Jeramiah J. Smith, Stephen R. Voss

**Affiliations:** 1 Department of Neuroscience, Spinal Cord and Brain Injury Research Center, and Ambystoma Genetic Stock Center, University of Kentucky, Lexington, KY 40536, USA; 2 Indiana University School of Medicine, Indianapolis, IN 46202, USA; 3 Independent Researcher, Vevay, IN 47043, USA; 4 Department of Biology, University of Kentucky, Lexington, KY 40506, USA

**Keywords:** genetic linkage analysis, axolotl, mutant, pigmentation, neural crest

## Abstract

The great diversity of color patterns observed among amphibians is largely explained by the differentiation of relatively few pigment cell types during development. Mexican axolotls present a variety of color phenotypes that span the continuum from leucistic to highly melanistic. The *melanoid* axolotl is a Mendelian variant characterized by large numbers of melanophores, proportionally fewer xanthophores, and no iridophores. Early studies of *melanoid* were influential in developing the single-origin hypothesis of pigment cell development, wherein it has been proposed that all three pigment cell types derive from a common progenitor cell, with pigment metabolites playing potential roles in directing the development of organelles that define different pigment cell types. Specifically, these studies identified xanthine dehydrogenase (XDH) activity as a mechanism for the permissive differentiation of melanophores at the expense of xanthophores and iridophores. We used bulked segregant RNA-Seq to screen the axolotl genome for *melanoid* candidate genes and identify the associated locus. Dissimilar frequencies of single-nucleotide polymorphisms were identified between pooled RNA samples of wild-type and *melanoid* siblings for a region on chromosome 14q. This region contains *gephyrin* (*Gphn*), an enzyme that catalyzes the synthesis of the molybdenum cofactor that is required for XDH activity, and *leukocyte tyrosine kinase* (*Ltk*), a cell surface signaling receptor that is required for iridophore differentiation in zebrafish. Wild-type *Ltk* crispants present similar pigment phenotypes to *melanoid*, strongly implicating *Ltk* as the *melanoid* locus. In concert with recent findings in zebrafish, our results support the idea of direct fate specification of pigment cells and, more generally, the single-origin hypothesis of pigment cell development.

## 1. Introduction

**Figure 1:**
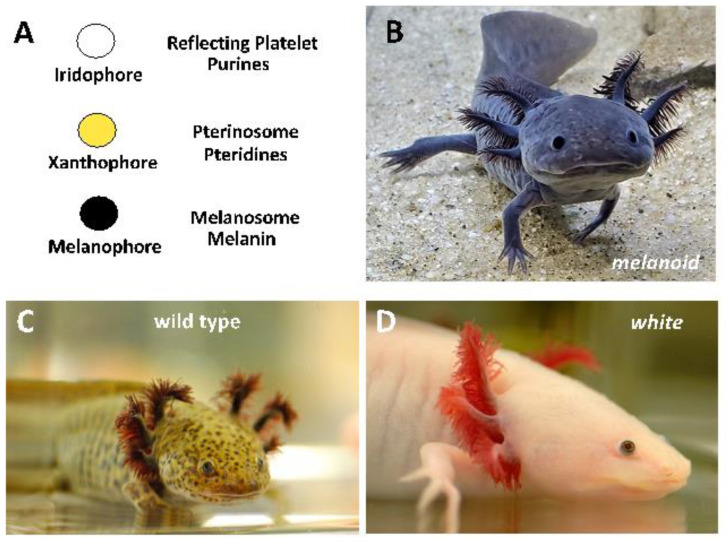
(**A**) The relative numbers of pigment cell types explain axolotl color variants. Iridophores have reflecting platelet organelles that synthesize purines. Xanthophores have pterinosome organelles that synthesize pteridines. Melanophores have melanosomes that synthesize melanin. (**B**) *Melanoid* axolotls have large numbers of melanophores, relatively fewer xanthophores, and no iridophores. (**C**) Wild-type axolotls have melanophores, xanthophores, and iridophores. (**D**) *White* axolotls have few to no melanophores and xanthophores and iridophores only in the iris of the eye.

The Mexican axolotl has a deep and colorful history. Thirty-three axolotls were famously imported from Mexico to Europe in 1863 to establish a population at the Jardin des Plantes in Paris [1]. Descendants from this population were distributed throughout the world to found laboratory populations that remain in existence today. All of the original Paris axolotls, except for one, presented approximately olive-green wild-type axolotl coloration, owing to the intermingling of black melanophores, yellow xanthophores, and more sparsely distributed iridescent iridophores (Figure 1). The lone non-wild-type axolotl in the original Paris collection presented a leucistic phenotype (*white*) characterized by few to no melanophores. *White* axolotls proved to be valuable in early embryological studies because they provided a non-pigmented background to monitor the behaviors of grafted, melanin-containing cells from wild-type axolotl donors. Today, *white* axolotls are preferred over more darkly pigmented wild-type axolotls for visualization of fluorescent probes and making transgenics with fluorescent reporter constructs. While the *white* phenotype was shown to be determined by a Mendelian locus over 100 years ago [2], the underlying lesion was only recently identified as a splicing defect in the *Endothelin 3* gene [3].

**Figure 2:**
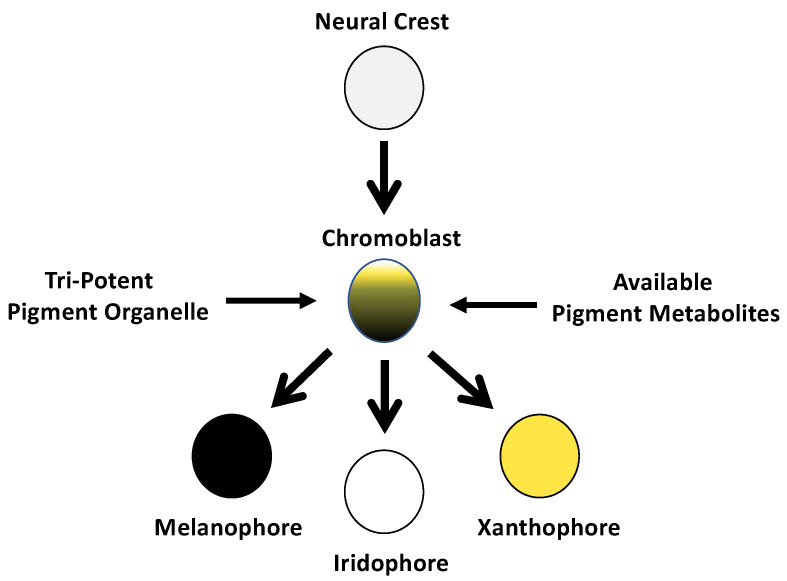
Schematic depiction of the single-origin hypothesis for pigment cells originally proposed by Bagnara et al. [8]. The three primary pigment cell types arise directly from a neural-crest-derived progenitor cell (chromoblast) with a tripotent pigment organelle. Fate specification of the chromoblast is determined by genetic and environmental factors that affect the formation of pigment-producing organelles. According to this hypothesis, melanophores permissively differentiate in *melanoid* axolotls because multipotent pigment progenitor cells have reduced XDH activity and lower levels of purine and pterin pigments that are synthesized by organelles of iridophores and xanthophores, respectively.

During the long domestication history of laboratory axolotls, additional pigment variants were discovered and shown to have simple Mendelian modes of inheritance. For example, the *melanoid* color variant was discovered in the 1960s from crosses that paired second-generation descendants of imported axolotls from Mexico [4]. Thus, like *white*, *melanoid* is a natural color variant that may still be present in the native Xochimilco, Mexico population. Relative to the wild type, *melanoid* is characterized by increased numbers of melanophores, fewer xanthophores, and an absence of iridophores. During development, melanophore numbers greatly increase in *melanoid* embryos and larvae as they age and become distributed throughout the skin to yield a uniform dark coloration that largely masks the yellow pigment of xanthophores. Soon after the discovery of *melanoid*, embryo grafting experiments [4,5] established that the effects of the *melanoid* mutation are intrinsic to the neural crest and therefore may be associated with a gene that regulates the differentiation of pigment cell progenitors. Subsequent experiments conducted by Bagnara et al. [6] showed that the dark *melanoid* coloration could be partially phenocopied by treating embryos (and extending treatments into the early larval period) with allopurinol, a xanthine dehydrogenase (XDH) inhibitor. Bagnara et al. [6] proposed that *melanoid* was associated with a defect in XDH activity, and Thorsteinsdottir and Frost [7] provided biochemical data showing similar concentrations of pterin pigment products of XDH activity in the skin of *melanoid*- and allopurinol-treated wild-type axolotls. These results were influential in motivating a hypothesis [8] that pigment cells differentiate directly from a common progenitor stem cell that has the potential to develop any of the organelles that define distinct or mosaic pigment cell types (Figure 2). This hypothesis proposes that genetic and environmental factors, such as the relative concentrations of intracellular pigment molecules present at the time of pigment cell differentiation bias the formation of pigment-producing organelles. According to this hypothesis, melanophores permissively differentiate in *melanoid* axolotls because multipotent pigment progenitor cells have reduced XDH activity and lower levels of purine and pterin pigments that are synthesized by organelles of iridophores and xanthophores, respectively [8].

Since the proposition of a single-origin basis for pigment cells, pigment cell differentiation has been studied intensively using teleost fish, especially the zebrafish. Many genes have been identified that function in the specification of pigment cell fates, such as the requirement of *melanocyte inducing transcription factor* (*Mitf*) for melanophores [9] and *leukocyte tyrosine kinase* (*Ltk*) for iridophores [10]. In contrast to the single-origin model of pigment cells, studies of zebrafish have revealed a more progressive process of differentiation involving intermediate progenitor cells that arise from a neural-crest-derived progenitor. For example, a bipotent precursor has been proposed for the origin of melanoblast and iridoblast intermediate progenitors that, in turn, originate melanophores and iridophores, respectively [10]. The overall process of progressive fate specification is largely controlled by genetic factors that act autonomously within cells, although cell fate is also influenced by environmental factors, including temporal and spatial feedback received from the environment, migratory cues, and pigment cell–cell interactions [11,12,13,14]. A cyclical fate restriction hypothesis was recently advanced to reconcile patterns of gene expression within pigment cell progenitor populations that are unexpected under a strict model of progressive fate specification [15]. A more recent analysis using techniques to accurately measure lowly expressed genes during zebrafish neural crest and pigment cell differentiation provide support for the hypothesis and direct fate specification of at least some pigment cell types [16].

The large axolotl genome presents a challenge to mapping genes for mutant phenotypes. Genetic linkage analysis provides an efficient approach for identifying genes that cosegregate with Mendelian phenotypes when prior biological knowledge is available to prioritize a few candidate genes to test [3,17]. However, when biological knowledge is lacking to prioritize a shortlist of candidate genes, such as in the case of *melanoid*, it is more efficient to pursue approaches that can access and evaluate regions throughout the genome for candidate genes [18]. In this study, we used bulked segregant RNA-Seq (BSR-Seq) [18] to identify two candidate genes for *melanoid*: *Ltk* and *gephyrin* (*Gphn*). Using CRISPR-Cas9 genome editing, we show that *Ltk* crispants are phenotypically similar to *melanoid*, strongly implicating *Ltk* as the *melanoid* locus. We discuss these results within the contexts of the single-origin of pigment cells hypothesis and teleost-inspired models of pigment cell differentiation.

## 2. Materials and Methods

### 2.1. Animal Procedures

Embryos, larvae, and adults (RRID: AGSC_100E, AGSC_101E, and AGSC_102E) used in this study were obtained from the Ambystoma Genetic Stock Center (RRID:SCR_006372), and all experiments were performed using either 50% (pre-hatching) or 100% (post hatching) rearing water (ARW: 1.75 g NaCl, 100 mg MgSO_4_, 50 mg CaCl_2_, and 25 mg KCl per liter buffered with NaHCO_3_ to pH 7.3–7.5) in a room maintained at 17–18 °C. Larvae were housed in glass or polypropylene bowls at either low density (8–10 per bowl) or one per container. After larvae reached 5 cm, they were transferred to 9 L boxes in an Aquatic Enterprises recirculating system. Larvae were initially fed newly hatched brine shrimp until they reached 3 cm total body length, then transitioned to California black worms (J.F. Enterprises, Linden, CA, USA) until they were large enough to be fed soft moist pellets (Wilbur-Ellis Nutrition, LLC, Vancouver, WA, USA). Animals were anesthetized using a 0.02% benzocaine solution. Benzocaine was first dissolved in 4 mL 100% EtOH; then, the chemical and solvent were diluted in 1 L of ARW. Animal care procedures were approved under University of Kentucky IACUC protocol 2017-2580.

### 2.2. Bulked Segregant RNA-Seq

A spawn was made using a wild-type female carrier of the melanoid allele and a melanoid male. A total of 16 melanoid and 16 wild-type siblings were reared to approximately 3 cm, and tail tissue was collected from each while under benzocaine anesthesia. The resulting tail tips were pooled into separate 1.7 mL plastic tubes and flash frozen with dry ice to create melanoid and wild-type bulk pools for RNA isolation and bulked segregant RNA-Seq [19]. Tail tissue samples were dissociated with 23- and 26-gauge needles, and RNA was isolated with TRIzol, then further purified using a QIAGEN RNeasy Mini Kit with DNase treatment (QIAGEN, Germantown, MD, USA). The resulting RNA pools were used to generate outsourced Poly (A) RNA-seq libraries that were sequenced on an Illumina HiSeq 2500 (Illumina, San Diego, CA, USA) by Singulomics (V4 sequencing chemistry, 125 bp paired end reads, Singulomics, Bronx, NY, USA). Reads from each pool of *melanoid* or wild-type individuals were mapped to the axolotl genome assembly [20] using HiSat2 [21]. SNPs were identified using BCFtools [22,23], and significantly different SNP frequencies at polymorphic sites were identified using Popoolation2 [24] and Fishers exact test. RNA sequence data are available from the Short Read Archive at the National Center for Biotechnology Information (Bioproject Accession PRJNA954746; SRR24142135 and SRR24142135).

### 2.3. Genetic Linkage Analysis

A spawn was made using a wild-type female carrier of the melanoid allele and a melanoid male. A total of 48 melanoid and 48 wild-type offspring from this spawn were reared to approximately 3 cm, and tail tissue was collected from each while under benzocaine anesthesia. During animal rearing, larvae were fed brine shrimp. The resulting tail tips were placed into separate 1.7 mL plastic tubes and placed on ice for DNA isolation. DNA isolations were performed using a New England Biolabs (NEB, Ipswich, MA, USA) Monarch genomic DNA isolation kit. DNA concentrations were determined using a Nanodrop (Thermo Scientific, Waltham, MA, USA), and all samples were diluted to 30 ng/uL for PCR. Using 150 ng per PCR reaction, the following conditions were used: 1 cycle at 94 °C for 3 m; 34 cycles of 94 °C for 45 s, 60 °C for 45 s, and 72 °C for 30 s; and 1 cycle at 72 °C for 7 min. PCR primers were designed to amplify amplicons for *Gphn* and *Ltk* loci (Supplemental File S1). PCR amplicons were treated with NEB ExoSAP-it and shipped to Eurofins for Sanger sequencing. The resulting sequences were compared using Geneious Prime software (Geneious Prime^®^ 2023.0.1, Biomatters Ltd, Aukland, NZ) to search for polymorphisms that could be used to infer linkage of *melanoid* to *Gphn* and *Ltk* loci.

### 2.4. CRISPR-CAS9 Disruption of Ltk and Gphn

Two guide RNAs (gRNAs) (CACGACCATCAAATCCGCGT and CTCATGCAATCGACCTCTTA) were designed to target the first and seventh exons of *Gphn*, respectively (Am2.2 transcript assembly, AC_02200042067.1, https://www.axolotl-omics.org, accessed on 5 January 2022). Additionally, two gRNAs (AATGGATGTATATTCCCCAC and GAATCTTCTTGTTCAACCGT) were designed to target the third and last exons of *Ltk* (a full-length *Ltk* gene model was built by merging AMEX60DD301011957.6 and AMEX60DD301011957.7 from AmexT_v47 transcriptome assembly, https://www.axolotl-omics.org, accessed on 5 January 2022). To perform CRISPR, gRNAs were duplexed with common Alt-R tracrRNA, aliquoted, and stored at −80 °C. All RNA products were synthesized by Integrated DNA Technologies. Guide RNAs and Alt-R tracrRNA were mixed with Cas9 ribonucleoprotein and injected into 1-cell stage embryos as described previously [25]. Tail tip tissue was collected from presumptive crispants for DNA isolation, PCR, and DNA sequencing according to methods described above.

### 2.5. Ltk Quantitative PCR

*Melanoid* and wild-type (axolotls not carrying *melanoid* alleles) embryos were sampled from two separate spawns at developmental stages 31, 36, and 43 [26]. RNA was isolated for 3 individuals per spawn at each developmental stage according to the method described above. Equivalent amounts of RNA were used to make cDNA using a ProtoScript II first-strand cDNA synthesis kit (NEB, Ipswich, MA, USA); then, 1 μL of the resulting cDNA was used for quantitative PCR (qPCR) using FastStart SYBR Green Master (Roche Diagnostics, Indianapolis, IN, USA). qPCR was performed on a Roche Lightcycler 96 using *Ltk* primers that were designed to generate a cDNA amplicon spanning exons 27 and 28. *Ltk* amplification was compared to a reference gene (*Atp5pb*) wherein primers were designed to generate a cDNA amplicon spanning exons 5 and 6. The qPCR primer sequences are listed in Supplemental File S1. The 2^−ΔΔCT^ method was used to calculate relative gene expression [27].

### 2.6. Ltk Genomic Sequencing

PCR primers were designed to amplify *Ltk* exons using genomic DNA samples prepared from a melanoid and a wild-type sibling. PCR primers are detailed in Supplemental File S1, and *Ambystoma mexicanum* nucleotide and protein sequences are detailed in Supplemental Files S2 and S3.

## 3. Results

### 3.1. Bulk Segregant RNA-Seq Identified Two Candidate Genes for Melanoid

**Figure 3:**
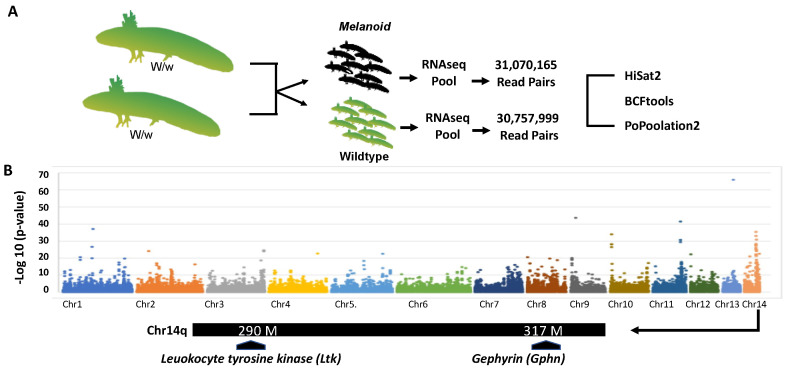
Overview of the bulk segregant RNA-Seq approach used to identify candidate genes for *melanoid*. (**A**) RNA was isolated for wild-type and *melanoid* siblings, and >30 m read pairs were generated for each pool. Sequences were aligned to the axolotl genome and used to identify a region of Chr14q that was differentially enriched for SNPs between the pools. (**B**) Map of a Chr14q genomic region showing the positions of *Ltk* and *Gphn*. There are at least 70 predicted genes within the interval defined by these two genes.

To identify loci linked to the *melanoid* locus, we used bulk segregant RNA-Seq [19] (Figure 3A). Embryos from a cross that segregated *melanoid* and wild-type individuals were used to create two *melanoid* and two wild-type RNA pools (N = 16 per pool) that were each subjected to Illumina short-read RNA sequencing to identify single-nucleotide polymorphisms (SNPs). Analysis of SNPs across the genome revealed a region on chromosome 14q with dissimilar frequencies between the pools (Figure 3B). Examination of genes within this region identified two candidates: leukocyte tyrosine kinase (*Ltk*) and gephyrin (*Gphn*) (Figure 3B).

**Figure 4:**
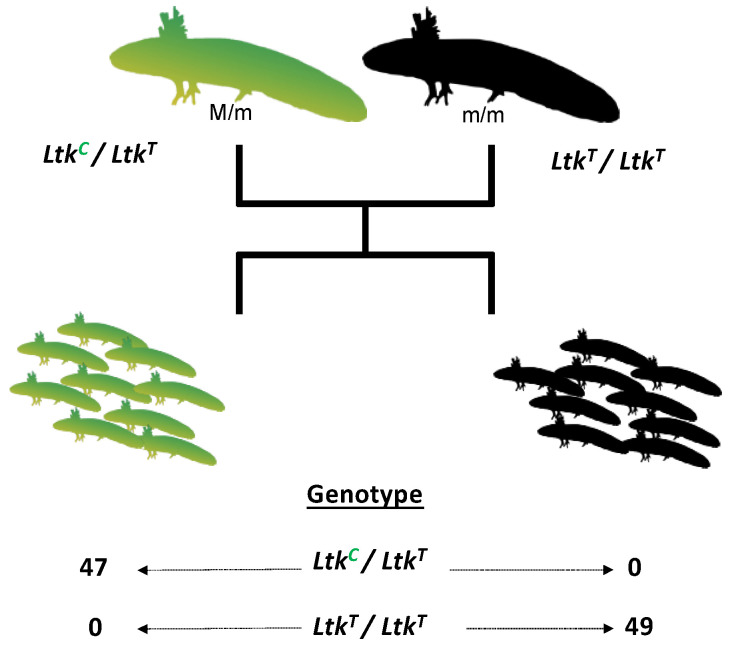
Genetic linkage analysis of *melanoid* and *Ltk*. An SNP within an *Ltk* exon was identified between a female *melanoid* carrier and a male *melanoid* (14q: 291,079,992, V6 Assembly). All embryos that inherited a heterozygous SNP genotype presented wild-type coloration, and all embryos that inherited a homozygous SNP genotype presented *melanoid* coloration, consistent with linkage to a single locus.

*Ltk* is the genetic determinant of the zebrafish *shady* mutant, which lacks iridophores [10]. *Gphn* encodes an enzyme that catalyzes the synthesis of the molybdenum cofactor that is required for XDH activity [28]. SNPs were identified within the *Ltk* coding sequence between an adult female carrier of *melanoid* and an adult *melanoid* male (Figure 4). These individuals were crossed to generate 96 embryos that segregated wild-type and *melanoid* phenotypes. All embryos that inherited a heterozygous SNP *Ltk* genotype at 14q:291,079,992 presented wild-type coloration, and all embryos that inherited a homozygous SNP genotype presented *melanoid* coloration, consistent with linkage to a single locus. DNA sequencing of the *Gphn* locus failed to identify an SNP that was informative for mapping; thus, we were unable to formally exclude *Gphn* as contributing to *melanoid* coloration.

### 3.2. CRISPR-Cas9 Disruption of Ltk Phenocopied Melanoid

**Figure 5:**
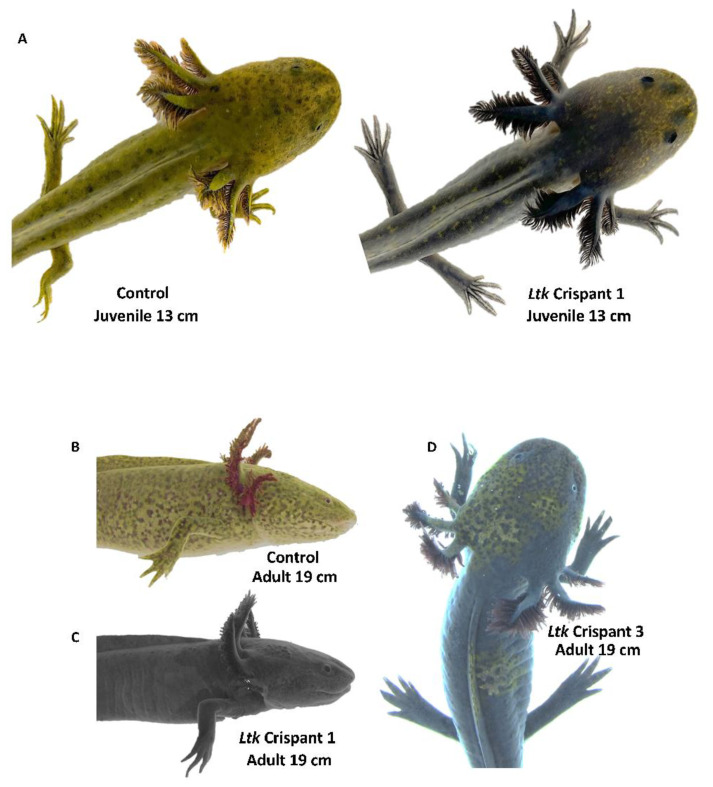
CRISPR-Cas9 editing of *Ltk*. (**A**) Examples of control non-injected and gRNA-injected (*Ltk* Crispant 1) individuals at the juvenile stage. (**B**) Adult control wild type with melanophores, xanthophores, and iridophore-pigmented eyes. (**C**) Adult *Ltk* Crispant 1 with melanophores masking xanthophores and no iridophores in the eye. (**D**) Adult *Ltk* Crispant 3 with melanophores, patches of xanthophores, and iridophore-pigmented right eye.

CRISPR-Cas9 was performed to determine if *Ltk* or *Gphn* gene editing would generate offspring with a melanoid-like color pattern. Two guide RNAs targeting *Ltk* and *Gphn* coding sequences were injected into one-cell-stage wild-type embryos and visually assessed for color at developmental stage 42 [26]. Approximately 150 embryos were injected for each gene, yielding 50 *Ltk* and 20 *Ltk Gphn* crispants that developed normally to hatching. No iridophores and relatively more melanophores were observed for *Ltk* gRNA-injected embryos than non-injected wild-type control embryos at developmental stage 42 (Supplemental File S2). The five most darkly pigmented *Ltk* crispants were assessed for *Ltk* genome editing. DNA sequence electropherograms for all five individuals yielded overlapping sequence traces with insertion/deletion polymorphisms consistent with CRISPR-Cas9 editing (Supplemental Files S3 and S4); these individuals were reared to larval and juvenile stages to assess fully developed color patterns. We note that two control embryos were heterozygous for a nucleotide site within the 20-base-pair gRNA2 target sequence. As both wild-type alleles would not be accessible for gRNA2 editing in heterozygous individuals, pigmentation phenotypes may be entirely attributable to *Ltk* gRNA1 editing. At 105 days post injection, the 5 *Ltk* crispants lacked iridophores in the iris and were characterized by a uniform distribution of melanophores throughout the skin that masked underlying xanthophores (Figure 5A,C). At 10 months of age, four of five *Ltk* crispants were indistinguishable from *melanoid* axolotls; *Ltk* crispant 3 presented patches of xanthophores on the head and flank and one iridophore-pigmented eye, consistent with CRISPR-Cas9 mosaicism (Figure 5D). In contrast to *Ltk* crispants, *Gphn* crispants presented wild-type color at developmental stage 42 and were significantly smaller than non-injected controls 110 days post fertilization. DNA sequence electropherograms for all nine individuals yielded overlapping sequence traces with insertion/deletion polymorphisms consistent with CRISPR-Cas9 editing (Supplemental Files S5 and S6); these individuals were further reared to assess fully developed color patterns. All 9 *Gphn* Crispants that were raised to 8 months of age presented wild-type coloration and catatonic states, resting on their back or sides instead of their limbs and only mounting a feeble swimming response when startled (Movie S1). These phenotypes are characteristic of molybdenum cofactor deficiency, complementation group C, a human disease that is associated with *Gphn* lesions [29]. These results implicate *Ltk* and exclude *Gphn* as the gene associated with *melanoid*.

### 3.3. DNA Sequencing of Ltk Exons and Quantitative PCR Analysis

The CRISPR-Cas9 *Ltk* editing results suggested the possibility that *melanoid* may be associated with a DNA mutation in the *Ltk* coding sequence. To test this hypothesis, we sequenced presumptive *Ltk* exons using genomic DNA samples from *melanoid* and wild-type siblings. As the wild-type sibling was a heterozygous carrier of *melanoid*, we were able to infer *Ltk* sequences for wild-type and *melanoid* alleles. A full-length *Ltk* gene model is not available in the current axolotl assembly (AmexG.v6). To generate a full-length model, we merged partial gene models (AMEX60DD301011957.6 and AMEX60DD301011957.7) to derive an *Ltk* model with 28 exons, 4320 nucleotides, and 1439 amino acids (Supplemental Files S6 and S7). PCR amplicons for the 28 exons were aligned to identify 19 single-nucleotide polymorphisms between wild-type and *melanoid Ltk* alleles. Most of the substitutions are predicted to be synonymous; however, seven nonsynonymous substitutions were observed. We aligned the wild-type and *melanoid* sequences relative to *Ltk* sequences from *Gallus gallus* (GenBank XP_046775648.1), *Xenopus tropicalis* (GenBank XP_00293378.2), *Danio rerio* (GenBank XP_005158843), *Pluerodeles watl* (GenBank KAJ1104026.1), and *Geotrypetes seraphini* (XP_033809083.1) (Supplemental File S7). We observed interspecific amino acid polymorphisms for five of seven nonsynonymous substitutions identified between the wild-type and melanoid alleles. Two nonsynonymous substitutions were unique for the melanoid allele relative to the other sequences: a serine (S) in place of a phenylalanine (F) at position 272 and an alanine (A) in place of a valine (V) at position 664. Whereas a V -> A substitution is predicted to be relatively conservative in regard to disrupting protein structure, owing to the non-reactive nature of alanine, an F -> S substitution is predicted to be more disruptive because these amino acids differ in hydrophobicity. Moreover, the F->S substitution is observed in the MAM (meprin, A-5 protein, and receptor protein tyrosine phosphatase mu) catalytic domain of LTK, an extracellular domain that mediates ligand binding and cell signaling in tyrosine phosphatase family proteins [30].

**Figure 6:**
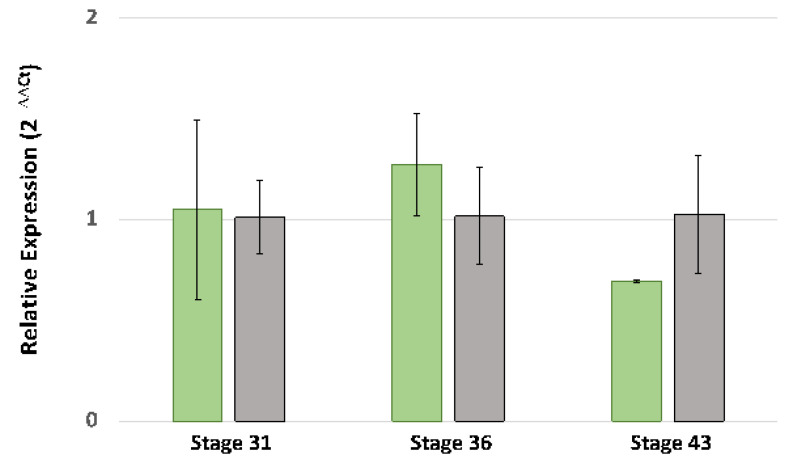
Quantitative PCR analysis of *Ltk* gene expression in A. *mexicanum* embryos (N = 3 wild-type and 3 *melanoid* embryos per stage). Green bars correspond to wild-type embryos; gray bars correspond to *melanoid* embryos. The error bars are standard deviations of relative fold expression. *Ltk* gene expression was not statistically different between wild-type and melanoid embryos for any of the stages (*t*-test *p* > 0.05).

In addition to structural variations, *melanoid* and wild-type *Ltk* alleles may be transcribed differently. RNA-Seq reads were recovered for *Ltk* exons from both the wild-type and *melanoid* pools used in the bulk segregant RNA-Seq analysis, suggesting that *Ltk* is expressed by *melanoid* individuals. However, the depth of sequencing performed in the bulk segregant analysis was not sufficient to determine if there are quantitative differences in expression between wild-type and *melanoid* individuals. Quantitative PCR (qPCR) was performed to determine if expression differed between *melanoid* and wild-type individuals at embryonic stages preceding stages 31 and 36 and during the time that iridophores are first observed in the iris (stage 43). *Ltk* levels were relatively low at all stages, and fold expression was higher in wild-type individuals at stage 43 but not statistically significantly higher (*t*-test, *p* = 0.08) (Figure 6). While more *Ltk* transcripts are expected in stage 43 wild-type individuals that develop iridophores, we did not observe a difference in gene expression between wild type and *melanoid* embryos at earlier developmental stages when iridophores are presumably differentiating. The bulk segregant RNA-Seq and qPCR results suggest that *melanoid* is not associated with altered *Ltk* transcription. Tentatively, *melanoid* may be associated with altered protein function, as we detected a non-conservative amino acid substitution in the *Ltk melanoid* allele that is not observed in any other vertebrate orthologous sequence.

## 4. Discussion

Many biologically significant mutants have been discovered during the 150-year domestication history of the laboratory axolotl [31]. These include several pigmentation mutants that affect quantitative differences in the relative numbers of three amphibian pigment cell types: black melanophores, iridescent iridophores, and yellow xanthophores [6]. Previously, we performed candidate gene linkage analyses to identify loci associated with *albino* (*Tyr*) and *white* (*Edn3*) [3]. Here, bulked segregant RNA-Seq was used to identify two candidate genes for *melanoid*, which, in comparison to the wild type, is characterized by increased numbers of melanophores, fewer xanthophores, and an absence of iridophores. Linkage analysis and CRISPR-Cas9 genome editing strongly implicate *Ltk* as the *melanoid* locus. Our study shows the utility of bulked segregant RNA-Seq for identifying Mendelian loci in the large axolotl genome and the efficiency of CRISPR-Cas9 to evaluate candidate genes in the axolotl. Although additional work is needed to identify the underlying *Ltk* mutation, which may be associated with a non-conservative amino acid substitution in the *Ltk* MAM protein domain, our results provide new insights about pigment cell fate specification in a representative amphibian.

**Figure 7:**
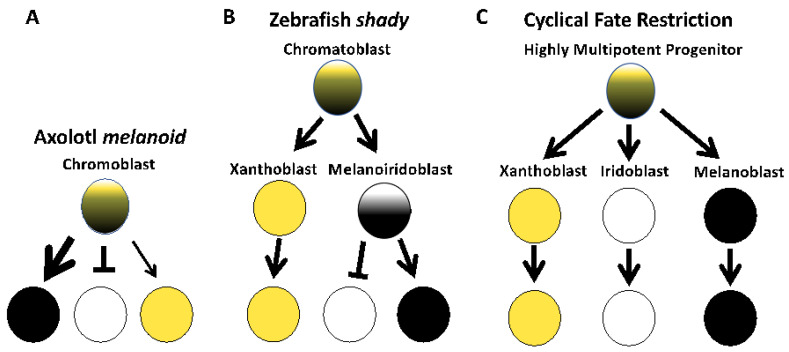
Models of pigment cell fate specification based on the results of this study and studies of zebrafish. (**A**) In axolotl, gene editing of *Ltk* affects iridophore and melanophore abundances, and proportionally fewer xanthophores are characteristic of *melanoid*. Arrows are weighted differently to show differences in pigment cell abundances. This model is consistent with direct fate specification of pigment cells from a multipotent progenitor. (**B**) In zebrafish *shady*, *Ltk* knockout only affects the specification of iridophores and does not affect melanophore or xanthophore abundances, consistent with progressive fate specification of iridophores and xanthophores [31]. (**C**) A cyclical fate restriction model recently proposed for zebrafish [15,16] posits dynamic, direct fate specification from highly multipotent progenitor cells. Yellow circles denote xanthophore lineage cells, white circles denote iridophore lineage cells, and black circles denote melanophore lineage cells.

Early studies of the *melanoid* axolotl color variant were influential in motivating the single-progenitor cell origin of pigment cells [8]. This hypothesis proposes that the three primary pigment cell types arise directly from a multipotent progenitor cell, and the relative contributions of genes and environmental factors that act within this progenitor cell population determine the relative number and abundance of the three pigment cell types. Consistent with this model, our results associate cell fate changes in all three pigment cell populations with *Ltk* gene function (Figure 7A). Mutagenesis of *Ltk* in axolotls similarly blocks fate specification of iridophores and affects the differentiation of melanophores. Melanophores were observed to uniformly cover the skin of axolotl *Ltk* Crispants and mask xanthophore yellow coloration, phenotypes characteristic of *melanoid*; these axolotls lacked iridophores in the iris. A formal analysis of xanthophore cell numbers is needed to verify that these cells are less abundant in *Ltk* crispants as they are in *melanoid* axolotls.

In zebrafish, *Ltk* is critical for the early development of iridophores. Specifically, in *shady* mutants that are associated with *Ltk* lesions [10] and in *Ltk* zebrafish knockouts [32,33], few to no iridophores are observed (Figure 7B). *Ltk* lesions specifically impact iridophores in zebrafish, inducing apoptosis, while melanophore and xanthophore populations are unaffected [10]. Our results suggest a broader role for *Ltk* in specifying axolotl pigment cell fates, showing that *Ltk* knockdown affects iridophore, melanophore, and, presumably, xanthophore populations, as is observed in *melanoid*. A lesion that simultaneously affects the fate of multiple cell types supports the idea of a multipotent progenitor and direct fate specification (Figure 7C), as has been recently proposed for zebrafish pigment cell differentiation [10,16] and as was proposed in the single-origin hypothesis for pigment cells [8]. Additional studies are needed to determine if there are fundamental differences within amphibia in terms of how key genes function to regulate pigment cell fates.

## Data Availability

RNA sequence data will be submitted to the Short Read Archive at the National Center for Biotechnology Information prior to publication.

## Supplementary Materials

The following supporting information can be downloaded at https://www.mdpi.com/article/10.3390/genes14040904/s1: Supplemental File S1: PCR primer sequences; Supplemental File S2: Representative control and *Ltk* gRNA injected embryos; Supplemental File S3: Electropherograms showing *Ltk* gRNA1 editing; Supplemental File S4: Electropherograms showing *Ltk* gRNA2 editing; Supplemental File S5: Electropherograms showing *Gphn* gRNA1 editing; Supplemental File S6: Electropherograms showing *Gphn* gRNA2 editing; Supplemental File S7. *Ltk* nucleotide sequences generated for *A. mexicanum* wild type and melanoid alleles; Supplemental File S8. Ltk amino acid sequences for A. mexicanum wildtype and melanoid alleles, and orthologous sequences from *Danio rerio*, *Gallus gallus*, Geotrypetes *seraphini*, and *Xenopus tropicalis*. Movie S1: All 9 Gphn Crispants that were raised to 8 months of age presented wild-type colora-tion and catatonic states, resting on their back or sides instead of their limbs and only mounting a feeble swimming response when startled.
