# Supplementary material for: Leukocyte Tyrosine Kinase (Ltk) Is the Mendelian Determinant of the Axolotl Melanoid Color Variant
Source: Genes (Basel). 2023 Apr 13;14(4):904. doi: 10.3390/genes14040904 (PMC10137446; doi:10.3390/genes14040904)
Supplement: Supplementary file 1 [file genes-14-00904-s001.zip › genes-2271687-supplementary.pdf]

Supplemental File S1. PCR primers that were used in the study to amplify *Gphn* and *Ltk* sequences for genetic linkage analysis, to amplify *Gphn* and *Ltk* sequences surrounding gRNA target sites, and to perform QPCR.

ID	Forward Primer	Reverse Primer
GPHN.1	AGTAGCTTTTAGTAGACCGCAGTAGG	CTGTTCAAATAAAAGGTATCCAGTCA
GPHN.2	AAGACTGAGCAGTATGTGGAGCTT	CGTATTTCAAAGATGAGAAAACACAA
GPHN.3	CAGCATTCATGTGGAAATTATTAAAG	ACTAAATCACCTCCTCTGACTGACAT
GPHN.4	TTCAGTTTATACTGCCAGCCCTAC	CTGTCCTTTTTCTCTTCTTCCTCTT
GPHN.5	CATTACAGTTAATGCATGCCTCTT	AACTCTCATCAGTCTGGTTTCTGAC
GPHN.6	TAGGCTGGCTGCCCAAAG	ATTCAGAAGTCATCCTGACAGCTC
GPHN.7	CTGCTGTGTGCGTGCATT	TAACACGTTTCTTTGTCTTATTACGG
GPHN gRNA 1	CTGCCAGGTAGTAAGTTGAATGTG	CGCTACCATATTTTACACCCTGTC
GPHN gRNA 2	CTCCCAACTAACCAGAGACTAACC	AACGAATAGAGACCAAAAATGCTT
LTK gRNA 1	TGAGCTTGTCTTGTACATCTTTCC	CATCAAAAGCAACAGATTCCATTA
LTK gRNA 2	ACAAAGACTGACCAACAAAAACAA	CTTAAGTCTTCAGAAAGGGACAGG
LTK Exon 1	TGTGCTAATGGACGTATGCTTTAT	TTGTGTCCTGTCTTTCTACGCTAC
LTK Exon 2	GAGAGAGAAGGAGCTATGAGCTGT	AATCGAAATGGGAGCTATTAACAC
LTK Exon 3	AGTCCATTTTGCTTTTAACCAGAG	ACTTCATGCGAGAAAATAGAAAGG
LTK Exon 4	AATCCCAATGAAGGCACGTAAC	ACTGGCAAACAAATACTGAGCTTT
LTK Exon 5	TGTAAATAGAACAGTCTGCCCTCA	CGAAGCTTTGTCGATTCTAATATG
LTK Exon 6	GCACACCTGTAGCCTAAAGAGTAA	GCAGTTGAGCAGTGTTATTTCCTA
LTK Exon 7	AGATATTTGGTCACATGGTTTTCC	GAGGCATCGGGTTATTAAGAAAT
LTK Exon 7-8	GCAGATGGTACTGCAAGAGCTTA	CTGCAGATTTACTGGAAACCAAC
LTK Exon 8.1	CTTTCCTACAGAACAGACCATTTAAG	ATTCAGCAAGGGAGAACTGGTC
LTK Exon 8.2	TCAAGTTTTCAGTTCACATTTTCG	CTGCAGATTTACTGGAAACCAAC
LTK Exon 8.3	GCATTTCTTAATAACCCGATGC	AACGTCAGAGGAGCACTGTGTA
LTK Exon 9	ACTATCACCTCCCACTTCTGTAGC	CGGCTGAAAATTGAGACTTTTATT
LTK Exon 10	CTGCTTGTGATGGTTCTCTAGCC	TATTCGCCATACACCCCTTATATT
LTK Exon 11	TATAGGAATCAATTTGCGCCTTG	TTCACAAGGTTAGAGGACAGACG

LTK Exon 12	TACAGAGTGAGAGAGGAGGTGATG	TTTACCCCCAAATAATACGACTGT
LTK Exon 13	TTGTGATTCACCCCTTTTATTTCT	CAAGAGAGCAGATGCAATGAGTAT
LTK Exon 14	GATTTGTTTTAGGACTTCCCCTTT	CTCTTAAGGCTAGCAAACAGTCGT
LTK Exon 15	GATCGCTTGGCTAACAATATAAGG	CGGTATCTGATCAAATTCAAAGAA
LTK Exon 16.1	CATTATCACCCACTCACACTCTGT	CTTTTAGAAGGTTGGGTCTTGGAG
LTK Exon 16.2	CAAGACCTAATGACTGTGTTTTGC	GGAAATGAACGAAGAAAGGTCTTA
LTK Exon 17.1	TGTTCATGTTCCCTAAGCATTAAA	TTTTCTTCCAAACAATACCAATGA
LTK Exon 17.2	GGGAAGGAGAGGAGACCCTTAC	CTGTACCTGTGCAGTTGATGTTGT
LTK Exon 18	GTAAGCGGTCGGAGTCTCTCTC	TATCCAGATTGCAGTGATAAAGGA
LTK Exon 19	CGAATTTGATATGTCAGTCTTTGC	CCTTAAGGGAAAAGTGAGATGTTT
LTK Exon 20	TTCCAAAAAGGTACTGCTAATTCC	ACCAAGTGAGTTGACAGCACTAAA
LTK Exon 21	GCGATATTATAATGGATGGGTCTT	GGGTTGATGGGAGATAGAGTAGAA
LTK Exon 22	TTCTCAGCAGTTCAGAATTGAATC	AGTTGGGGTTCTAAGATAGACACG
LTK Exon 23	GCTCTGCCATAGATGTAAAGAGGT	TGGGAGAACACAGAACTTTAATCA
LTK Exon 24	CAGTATTTTTAGAATGGGGCCTAA	AAACCCACAGTCTTACCTTTCTGA
LTK Exon 25	AAATATCTTGAGGGTGACAAGCTG	GGTGAGATCACTTTACGCATGTTA
LTK Exon 26	ATGCACGTGTTTCTTTTGTACATT	GCCAGTACCTTTGATTTACGAGTT
LTK Exon 27	TCTTGATCGTTCTTTGATGTATAAGG	GTGACTATTGATTGGCACATGAAG
LTK Exon 28.1	AAAGAATAGTGAGATTTCGGATGG	CGTCGGTTTAGGGTTAGTATTTTG
LTK Exon 28.2	CAAAGACTGACCAACAAAAACAAG	GGGATTACATTATCGACTCAATCC
LTK Exon 28.3	TACTTCACTAATTCCCAACGTAGC	CAATGTTGGATCTAAGAACCTTGA

LTK qPCR	GTATCGAATCATGACACAGTGTTG	ACAGGAAGCTCAGTATTGACGAC
ATP5PB	CTCAAGAGGTGAAAGATACTGCAA	CCAGTAGCATGACAACATTACTCC

Supplemental File S2. Representative control and *Ltk* gRNA injected embryos at developmental stage 42.

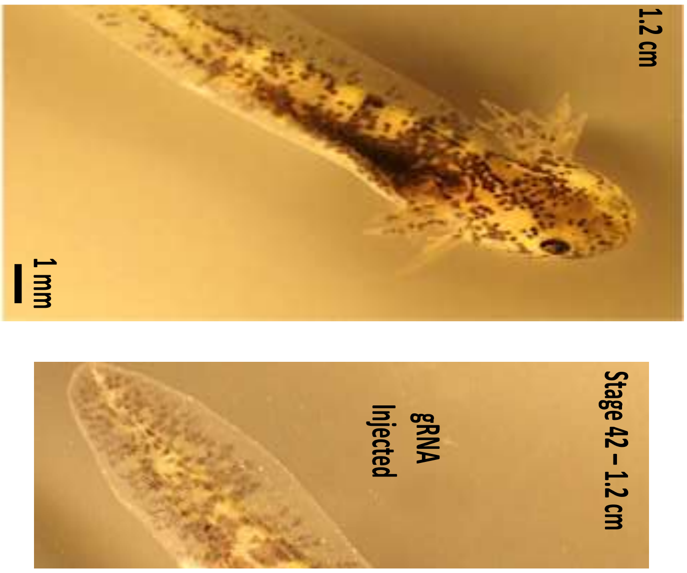

Supplemental File S3. Electropherograms showing *Ltk* gRNA1 editing. The sequences were generated using the *Ltk* gRNA1 reverse PCR primer.

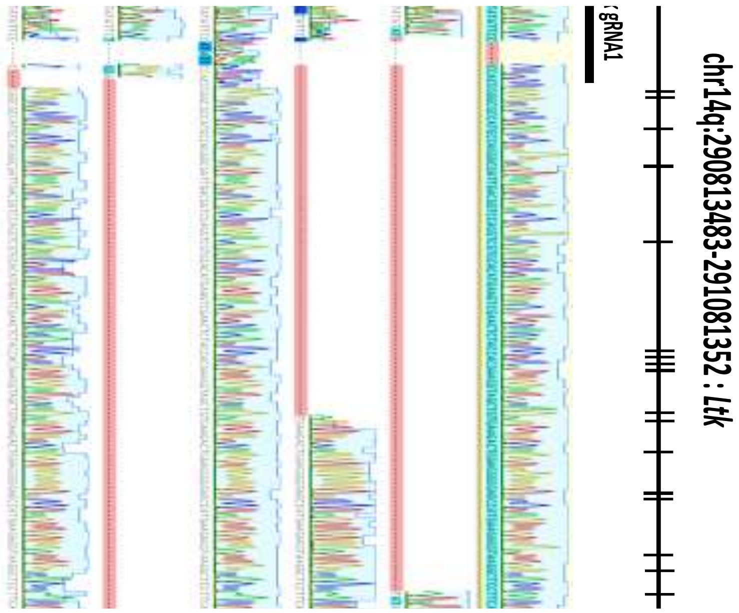

Supplemental File S4. Electropherograms showing *Ltk1* gRNA2 editing. The sequences were generated using the *Ltk* gRNA2 forward PCR primer.

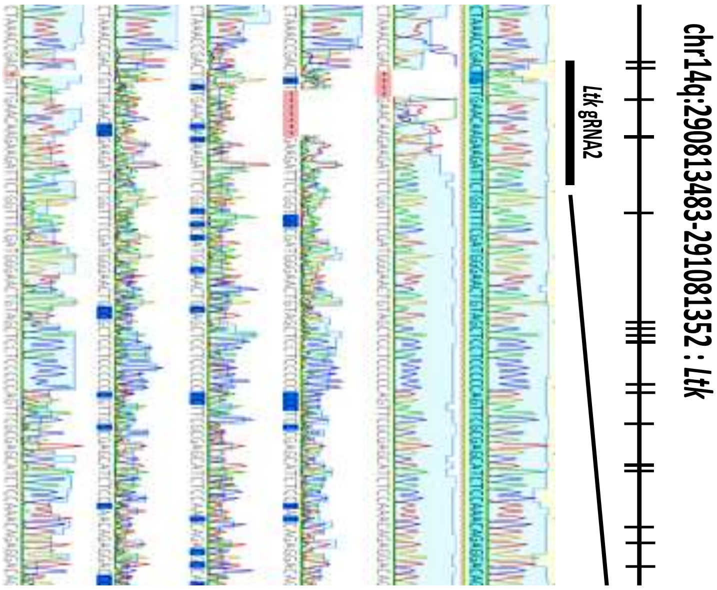

Supplemental File S5. Electropherograms showing *Gphn* gRNA1 editing for 5 of 9 Crispants that were assessed for genome editing. The sequences were generated using the *Gphn* gRNA1 forward PCR primer.

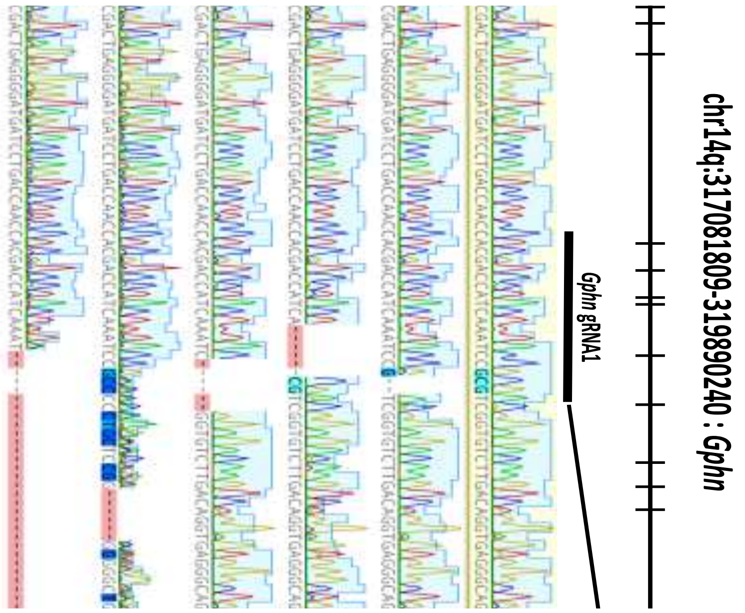

Supplemental File S6. Electropherograms showing *Gphn* gRNA2 editing for 5 of 9 Crispants that were assessed for genome editing. The sequences were generated using the *Gphn* gRNA2 forward PCR primer.

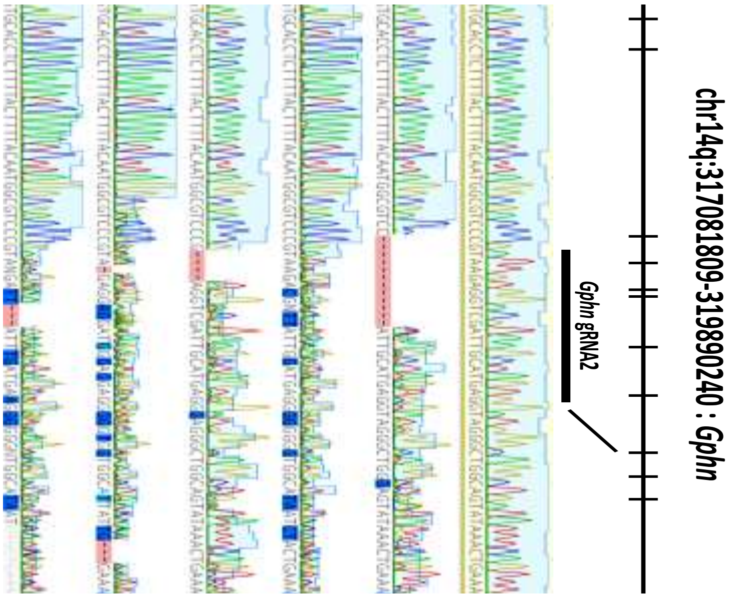

Supplemental File S7. *Ltk* nucleotide sequences generated for *A. mexicanum* wild type and melanoid alleles. Dots show conserved nucleotides.

```
Consensus           ATGTGCTTAGTTGCAGTTTCTGGCTTTGGATCCAACGCCATAGAAGACATCACATCGCCGAGGCCACCGTCGCCAGGCGCATCACAGCAGGCTGATGTAACACTAGAGGACCACCTGTCCAGTGC   125
Ltk_melanoid allele .............................................................................................................................   125
Ltk_wildtype allele .............................................................................................................................   125

Consensus           TGAGTACTCGTGCAACTTGGAGTCTGACTGCCCATGGACTTCTTCCAGGACGGATAACGCCGAGCCCCACTGGGAGCGGATACCACCGGGTGCCCTGACAGGACAGCATGAAGACCTCCTTCTGG   250
Ltk_melanoid allele .............................................................................................................................   250
Ltk_wildtype allele .............................................................................................................................   250

Consensus           GGGACAGCGCATCACCTGGCGATATGAAAGGTGGACTCCTTCTCCTAAACACTAGTCTACACGCAGGSCAATGTGAGTTCCGCCTGAGCAGCCCGCTCCTGCCCTGGAGCTCGGCTCAATGCCTG   375
Ltk_melanoid allele ...................................................................C.........................................................   375
Ltk_wildtype allele ...................................................................G.........................................................   375

Consensus           CTGGAAATGGATGTATATTCCCCACCGGGCGCCATGCCAGGGCGATTGACGGTCCAGGTCGTGCACATGAAGTCGAACTCTACCACGAAGGTAGCTGTGAAGACTGGAGGGGGAGCCATGAAGAG   500
Ltk_melanoid allele .............................................................................................................................   500
Ltk_wildtype allele .............................................................................................................................   500

Consensus           GGAGACCAGGAAGCTGTTGACGGCGGACATTGGCCAGGTGGACGCGCCGTTCCAGATCTCCGTGGCGTATTCATCGTGCGGCGTGGAAAAAGAGGAAGGCCTGCTGGCGTTCAACTCCCTGCGAC   625
Ltk_melanoid allele .............................................................................................................................   625
Ltk_wildtype allele .............................................................................................................................   625

Consensus           TCAGCAGCTGCTTCCGGGATGAAGAGGGTCCTGACCTGTCCAGGTGCACCCAGGACTCCTCGTTCTATTGCACATCAGGGGCCTGCATCAGTCGCCTGCAGGTGTGCGACTTTCATCTGGACTGT   750
Ltk_melanoid allele .............................................................................................................................   750
Ltk_wildtype allele .............................................................................................................................   750

Consensus           CCCGGGGGAGAGGATGAGGGCGATATTTGTGCATCCCTCCTCCCAGAGGGAGCATTTTGTTCCTYCGAGTCGGGAGCCTGCGACTGGGCTATGGACGATGCCGCSGCAACCGCGAGTTGGTCCAT   875
Ltk_melanoid allele ................................................................C.......................................C....................   875
Ltk_wildtype allele ................................................................T.......................................G....................   875

Consensus           CGTGGACTCGAAGCAGACGGATCCCGAAGCACCGGTTCTCGGCTCTGCTTTAAAAGCTACTGCTGGGCACTTCCTTTACCTGCGGGTACAGGATCAGTGGGCAGATGGTACTGCAAGAGCTTACA  1000
Ltk_melanoid allele ............................................................................................................................. 1000
Ltk_wildtype allele ............................................................................................................................. 1000

Consensus           GCGCCAACCTGCCAGGCGTGTTTTCCAGCCAAGATTGTCAGGTCAAGTTTTCAGTTCACATTTTCGGGGCTTACAATGGAACAGTCTCCCTCGCTGTCCTTGAGAAGGTGGCGGGCCCTACAGGC  1125
Ltk_melanoid allele ............................................................................................................................. 1125
Ltk_wildtype allele ............................................................................................................................. 1125

Consensus           AGTGCCCAGCTGGTTTGGGAAAGAACGGCCAGCTGGGGCGACCGTTGGTTTCCAGTAAATCTGCAGCTGCCAGAGCTCCGGGACGGGTTCCAGCTGGTGCTCCAGGCTTCCTGGGGTTGGGGCTC  1250
Ltk_melanoid allele ............................................................................................................................. 1250
Ltk_wildtype allele ............................................................................................................................. 1250

Consensus           GCTGGCGGACATCGCTGTCGATAACATCACCGTCACAGCAGATTGCTTCCACGAAGGAAGAAGACATGGTCAAGCTGGCAAACACCAGTTTGCTCGGGAGGTAAACCGAAGGGAAGAGCCACAGC  1375
Ltk_melanoid allele ............................................................................................................................. 1375
Ltk_wildtype allele ............................................................................................................................. 1375

Consensus           TGACCCCTTTGCCGGATCCCTCCGCTTCAGGGGCGACSTCAGACGTGGAGTGGTGGTTTACAGCGTGTGGCGCCAGTGGCCCCCGGGGGCCAACGCAAGCTCAGTGTGACAATGCTTATCGGAAC  1500
Ltk_melanoid allele .....................................C....................................................................................... 1500
Ltk_wildtype allele .....................................G....................................................................................... 1500

Consensus           ACCAACGTCTCGGTCACCATGGCAACGGACGGCGCACTCCAGGGGGTGCAAATATGGAGAGTGCCCGCAACTAACATCTACAGAATTTCTGCCTATGGCGCAGCTGGCGGAAAAGGACTGAAGAA  1625
Ltk_melanoid allele ............................................................................................................................. 1625
Ltk_wildtype allele ............................................................................................................................. 1625

Consensus           CCACAACAAGCGATCCCACGGTGTCTTCATATCCGCCGACTTCCGACTCCAGAAAGACGAACTCCTCTACATCTTGGTGGGACAGCAGGGGGAAGATGCTTGCCCAGGGGGAAATGTGCTGACTA  1750
Ltk_melanoid allele ............................................................................................................................. 1750
Ltk_wildtype allele ............................................................................................................................. 1750

Consensus           GGGAGCTCTGCCTCGGAGAGTCATCCATCACTGAATTGGAGTACTATGAKAATAAAACCCTTCTGCAGTGGGCTGGAGGAGGAGGTGGYGGTGGGGGAGCCTCCTACATATTTAAGCTCGACAAC  1875
Ltk_melanoid allele .................................................T......................................C.................................... 1875
Ltk_wildtype allele .................................................G......................................T.................................... 1875

Consensus           GGGGTCTTCAGGCCCCTACTCATCGCAGCTGGAGGAGGCGGGAAGGCCTACTTGGAAGACCCAAAGGGTTCCTCGGAACAAGCRCACTTGGAGGAGYTTGAGAAGAGCATTGCAGYCCCGGGTGT  2000
Ltk_melanoid allele ...................................................................................G............T..................C......... 2000
Ltk_wildtype allele ...................................................................................A............C..................T......... 2000

Consensus           CAATGGAAGAGCTGGGGCAGCAGGTGGYGGCGGTGGATGGAATGACAGCACCCTCCTACCCCGGTCTGGGAAGTCGCTCATGGAAGGGGCAGAGGGYGGTAAGGTTTGCCCCCAAGCATCAGCTA  2125
Ltk_melanoid allele ...........................T.....................................................................T............................ 2125
Ltk_wildtype allele ...........................C.....................................................................C............................ 2125

Consensus           ATCTGCATTGGACCACGTCGGGGGGATTTGGAGGCGGAGGGGGCGCCTGTACCTCTGGAGGTGGAGGTGGAGGGTATAAAGGTGGAMATGCATCAGAATTTGATGACCTCACAACCGATGGAGAA  2250
Ltk_melanoid allele ......................................................................................A...................................... 2250
Ltk_wildtype allele ......................................................................................C...................................... 2250

Consensus           GATGGCATCTCCTTCATGAGCCCAGCCGGGGAGATTTTTCTGCAGCCTCTGGCAGCCATGGAGAGCCATGGGGAGGTGCAGATCGAAGTGTTCTTGAACTGCAACCACTGTGTGTCTGGAAACTG  2375
Ltk_melanoid allele ............................................................................................................................. 2375
Ltk_wildtype allele ............................................................................................................................. 2375

Consensus           CAAGCGGGACCCCCGAACCAAGCTCGTCACCTGCATCTGCAAAAGTGGAGAGCTGCTGGCCAGTGACAACATCAACTGCACAGAGCCAGACTTGGAGGAGCACTTCCCCATCCCCCTTATCCTGT  2500
Ltk_melanoid allele ............................................................................................................................. 2500
Ltk_wildtype allele ............................................................................................................................. 2500

Consensus           CAGTGGTCACTGCGACGGTAATACTTGGTGTGATCTTCACTTGTGGCGGCCTGTCAGTTGTTTATCACCGCAAGAAGCAGCAGCTTGAGGGAGCACGTATTCGACTGCAAAGCTCAGAGTACAAA  2625
Ltk_melanoid allele ............................................................................................................................. 2625
Ltk_wildtype allele ............................................................................................................................. 2625

Consensus           CTGAGTAAAATCCGAACGTCCACTATCATGACAGACTACAACCCTAACTACTGCTTTGCGGGAAAGGCAGCTACATTAAGTGAGCTAAAGGAGATACCTCGCAAGAACATCTCCCTTCTGCGGGC  2750
Ltk_melanoid allele ............................................................................................................................. 2750
Ltk_wildtype allele ............................................................................................................................. 2750

Consensus           CCTGGGGCATGGTGCTTTTGGAGAGGTTTATGAGGGGACAGTGGTAGGGATGGCAGGTGACCCCAACCCTCTCCAGGTGGCAATCAAGACGCTGCCTGAAATCAGCTCAGAACAGGACGAGATGG  2875
Ltk_melanoid allele ............................................................................................................................. 2875
Ltk_wildtype allele ............................................................................................................................. 2875

Consensus           ACTTCCTCATGGAGGCCTTGATTATCAGTAAGTTCAGTCACCAGAACATTGTCCGCTGCGTCGGAGTGAGCCTGCAGACGCTGCCGCGCTTCATTCTGCTGGAGCTCATGGCTGGTGGAGACATG  3000
Ltk_melanoid allele ............................................................................................................................. 3000
Ltk_wildtype allele ............................................................................................................................. 3000

Consensus           AAGAGCTTCCTCCGACAGAATCGGCCTCGCGTGCTCAAGCCATCCAGCCTGACGATGCTGGACCTGCTAAACATGGCGCGAGATATTGCCTGTGGCAGTAAATACCTCGAGGAYAATCACTTCAT  3125
Ltk_melanoid allele .................................................................................................................C........... 3125
Ltk_wildtype allele .................................................................................................................T........... 3125

Consensus           CCACAGGGATATAGCAGCCAGAAACTGCCTTCTTACCTGCGGCGGCCAGGGTCGTGTGGCCAAAATAGGAGACTTTGGAATGGCTCGGGACATTTACAGGGCCAGCTACTACCGGAAAGGGGGCA  3250
Ltk_melanoid allele ............................................................................................................................. 3250
Ltk_wildtype allele ............................................................................................................................. 3250

Consensus           GGGCCATGCTTCCTGTCAAGTGGATGCCACCGGAAGCCTTCCTGGAAGGAATATTCACTTCAAAAACAGACACATGGTCCTTTGGCGTACTGCTTTGGGAGATCTTCTCCTTGGGTTATATGCCT  3375
Ltk_melanoid allele ............................................................................................................................. 3375
Ltk_wildtype allele ............................................................................................................................. 3375
```

```
Consensus            TATCCATGTAAAACCAACCAGGAAGTGCTGGAGTTTGTCACCAGTGGAGGGCGCATGGATCCCCCAAAGAATTGTCCCGGGCCTGTGTATCGAATCATGACACAGTGTTGGCAACACAGTCCGGA  3500
Ltk_melanoid allele .............................................................................................................................  3500
Ltk_wildtype allele .............................................................................................................................  3500

Consensus            ATATCGACCGGATTTCACCACCATCTTGGAAAGGATCAAGTATTGCACCCAGGATCCTGACGTCGTCAATACTGAGCTTCCTGTGGAATGCAGCCCAGCACCGGAAGAAGAGGGGAGTGCCGGRA  3625
Ltk_melanoid allele ...........................................................................................................................A.  3625
Ltk_wildtype allele ...........................................................................................................................G.  3625

Consensus            TGCGGCCCGCAGGCTCCAGTGGCATGACTCCTCTGGTGATGAGCCCCAGCCTCRCACAGCAGCTAGTACAGCGATCACCAGAGGCACAACGACTAGGGCCTAAAATTGTCGAGTGCCCGCAAGAG  3750
Ltk_melanoid allele .....................................................A.......................................................................  3750
Ltk_wildtype allele .....................................................G.......................................................................  3750

Consensus            CTCATTGTGGACAGCCTGGGAGGATGGGCTGTGCAGGAGCCATCGCTGCCGTGCCTCAGTGACTCCACCAGTGCGCCCTGGTTCCAGCAAAACTCYAGCTCAATTGCTGAGKGCACAMTGCAGAC  3875
Ltk_melanoid allele ...............................................................................................C...............T....C.......  3875
Ltk_wildtype allele ...............................................................................................T...............G....A.......  3875

Consensus            AGCACAAAGACTGACCAACAAAAACAAGAACCTCTGGAACCCCACCTATGGCTCCTTAGTCATGGATAGTGCCTGCCGATCTAGTCAAAATACTAACCCTAAACCGACKGTTGAACAAGAAGATT  4000
Ltk_melanoid allele ............................................................................................................G................  4000
Ltk_wildtype allele ............................................................................................................T................  4000

Consensus            CTGGTTTCGATGGGAACTGTAGCTCCTCCCCCAGTTCGCGAGCATCTCCAAACAGAGGACAGTGCGCTCACCTAGACATTGGCGGGATTGAGCTGGTCAAGCTCCAGAACTTTCCATGCGGCAAT  4125
Ltk_melanoid allele .............................................................................................................................  4125
Ltk_wildtype allele .............................................................................................................................  4125

Consensus            GTGAATTATGCATACGATGACCTCTGTTTTGAAATGGATGGGGCCTCTGCAGTGGACACAAGAGACAGAACAGCTGTCCTAAGAAGTGCCAGCCTCTGCGGCGAGGGCCTTGTTCTCCGAAAGCA  4250
Ltk_melanoid allele .............................................................................................................................  4250
Ltk_wildtype allele .............................................................................................................................  4250

Consensus            AGACAAACCACCTGGGGAGAGGGACTCCGGCCTGTCCCTTTCTGAAGACTTAAGTCTCACACCAGTATAG                                                        4320
Ltk_melanoid allele ......................................................................                                                       4320
Ltk_wildtype allele ......................................................................                                                       4320
```

Supplemental File S8. *Ltk* amino acid sequences for *A. mexicanum* wildtype and *melanoid* alleles, and orthologous sequences from *Danio rerio, Gallus gallus, Geotrypetes seraphini, and Xenopus tropicalis*. Dots show conserved amino acids relative to the consensus. Pink amino acids mark nonsynonymous polymorphisms between the *A. mexicanum melanoid* allele and *A. mexicanum* wildtype allele that are polymorphic in other vertebrate orthologs. Red amino acids mark nonsynonymous polymorphisms that are unique to the *A. mexicanum melanoid* allele.

```
Consensus             MGFPXXXJXXXXXXXXMCLVAVSXXGSXAIEDIXSPRPPSPGAXQXXDVXLEDHLSSXEXSCBLESDCPWTSSRTXNGXPXWXRISXGALTSQ-REXLLLXDSSSPGDMXGGILLL--NT   117
A. mexicanum melanoid ----------------.......GF..N.....T.........S.QA..T.......A.Y..N............D.AE.H.E..PP....G.-H.D...G..A.....K..L...--..   101
A. mexicanum wildtype ----------------.......GF..N.....T.........S.QA..T.......A.Y..N............D.AE.H.E..PP....G.-H.D...G..A.....K..L...--..   101
D. rerio              ----MDYITRQTFVKLALFIFTVLRS.C.LLEKAAED.VH.NPL.SSPAEDS.V-----SF.DF..P.S..L.SHST.G-D.FIT.AQQHR.SR.DTQPIR.Y.T-.KSE.HF...KPSS   109
G. gallus             -----------------MG.V..CS..HEVNSSAPIP.EKFSLIRNRM.SFLQP-.EP.I..IIG.S.S.MT.TDSF.K.D.VLS.VSV.V..E.QQ...EN...Q.GVE.D.F..--..   100
G. seraphini          ---------------------------------------------MSLTK.M.C.PHLSSALQ.ILWTITMLLVIICSGLDEKK..TVK.S.MS.NETVSY.KP.---NA......--.F    70
X. tropicalis         ....FSVLLVSHLLVWTH.L.DLCS.LH.ESIMSLNL.-----ITSES.SDDEKVEQLGQ..DFKIH...V..IKRD.K.H.VHTVV.E.A.EKQRE-..ESQ.T.RHKQDE..F.--..   112

Consensus             SXLXAGRCEFXLSSPXLPWSSAXCLLELXVYSPXG-PMPGRXTVQVVHMKSNSXTXIXLKTGGXEMKRXTWKLLTAXIGQVNEPFQISXXYSSCGVEXXXGLLAFBSLRLXNCFXDEEGP   236
A. mexicanum melanoid .-.H..Q...R....L......Q....MD....P.-A....L...........T.KVAV....GA...E.R.....D....DA.....VA.......KEE.....N....SS..R.....   219
A. mexicanum wildtype .-.H..Q...R....L......Q....MD....P.-A....L...........T.KVAV....GA...E.R.....D....DA.....VA.......KEE.....N....SS..R.....   219
D. rerio              .H.S....S.HMT..VVLS.GPF.H.Q.ARFQ.E--.HA.NISAF.K.TD.TDIKP.D.TIKEQ.SDSSQ.EV.E.V...L.....VTVQ..A.SSHEV-.F...D..E.K..VMGDDYV   226
G. gallus             .R.-PD..K.S.H..I..G.VDS.....A...QDAV.LLQ.F..EIRYVDT.RNII.S..V.HE.DAGVK..SMM.Q..RIER..K.TLL..N..AQEA-....VG..Q.R...H.K.N.   218
G. seraphini          .HF-SE....HFQ.HV..R...S.....FF..WHE-.I.E.F...IQ.L.T.LS.Y.P.RQE.E.WIKG.--PV.VQ........R..LL.....L.NST..M.LD....K...H..V.L   186
X. tropicalis         .QSTENCAVTW.L..I.TAG.EM.Q...VL..QED----AIM.AM.HNI.A..SLPLS..EK-TVN..SN..M.S.E.AG........LD.IH.-.QTADHPIVLE..T.RH.SAGYQDS   226

Consensus             DL-SRCTXXSSFXCTSGGCISXLQVCDFHXDCPXGEDEGDICASXLPEGXYCSFESGACGWTXBDTAAT-SWSIVDSKQXXXEAPXLGSALKATXGHFLYLRVQSQWADGXARAYSANLP   354
A. mexicanum melanoid ..-....QD...Y....A...R.......L...G..........L....AF..S.....D.AMD.A...A.........TDP...V........A.........D.....T.........   338
A. mexicanum wildtype ..-....QD...Y....A...R.......L...G..........L....AF........D.AMD.A...A.........TDP...V........A.........D.....T.........   338
D. rerio              ..G.D.EKY..LQ.H.....EKQR.....T...E.....L..ST-..L.S......L.S...LAA..QS--..RL.SGQ.LIEDTHL..TT..N.Q....F.K.RGHGDERE.LVQ.PA..   343
G. gallus             ..-.IYNKG...P.LH...V.H..I.E.IS....TK.V..VR.D.-.....SH......K.S....LNE...S-P...KGFNDMIHSGSFV....QS.G.......AD.NHTN.M.K.F.TS..   335
G. seraphini          HI-.P.KNGFD.P.MY....TH....N.YT...SE....T..MQR....A......A......LN....V-T..FK..ENIAHKI.F...S.Q..KN..........QE..E.S....R..   304
X. tropicalis         FM-PT.VGEN..F.DN.D..NYVNL...TN...T.....AL.EII..S.S......DD.T.....PY.GND.-T.LYT.AT.IEDTS.---.D..LVK......S...KRS..S.TI...VF.   341

Consensus             XVXSNQDCQVQFSVHIFGAYNGTVSLXVLEXXAG-TGSAQLVWERTGSWGDXWFPVTLXLPELQNXFQLXLQASWGWXSLADIAVDNITFXADCFHXGRRXXQ-XGKHQFAREVNRXEXP   472
A. mexicanum melanoid G.F.S.....K...............A...KV..P............A....R....N.Q....RDG...V.......G...........VT.....E...HG.-A...........R.E.   457
A. mexicanum wildtype G.F.S.....K...............A...KV..P............A....R....N.Q....RDG...V.......G...........VT.....E...HG.-A...........R.E.   457
D. rerio              STI......L...LYRY.DF....L.S.V.SG.S---APA.I...S.H.K.A.QEI..PIT.IL.G.H.KV..F.TSG.K....L.D.SLS.A..DTELNELL----.E-----GLPHDL   451
G. gallus             ASIATE.Y.I.....V..S....I.FS.R.ERN.-VPVTLPM...A....S.H..LI..PM.V...R...Q.L.T....N.Q....I.....GM...AD..QPTHFK.....P..ISSPDEH   454
G. seraphini          AMVA.NT.K...A...N.TL...S...FLV.ES.V-..KFW....KV..S..H..I..RP......R.H.K.L...S.N....V........GL...NDE..QID-SERQ.VSQKI.AL.D-   421
X. tropicalis         K.VT.NV..LS.KFL.S.I...SLGIYG..DGK.-M..SVKL.VKQ.AS.NF.NSG..QI.QF..R.R.LVV.T...N...H..L..V..SV....A..IEIG----YENHGL.DDF.DD   456

Consensus             QLTPLPEPSASGETSXVEWWFTXCGASGPHGPTQAQCDNAYRNTNVSVTVXKEGPLXGVQXWXVPATNXYRISAYGAAGGKGAKNHNKRSHGVFISAXFXLQKDEJLYILVGQQGEDACP   592
A. mexicanum melanoid ......D.....A..D......A......R....................MATD.A.Q...I.R.....I.............L..............D.R.....L.............   577
A. mexicanum wildtype ......D.....A..D......A......R....................MATD.A.Q...I.R.....I.............L..............D.R.....L.............   577
D. rerio              DFS........-.A.PIT....S......F........S.........V.G.....R...M.K.....T.K............................T.P.E.GDI....I.H.......   570
G. gallus             L.N..E...LP.D..V.L...ST..................GS....I.E...R.R...V.R.....R..................E........T.Q.E...L.............   574
G. seraphini          M.F.P.QS.TL..P.EM..L.SP...R.......V........M.G.....---...Q...M.K....DT.K..............P.........A.I.Q.....I.S.......G....   538
X. tropicalis         TVM..S...YTD.N.EL.LQ..S...........T.......SS.I..V.G.....R...L.K..F.SV.Q................NPA.......I.P.....I.............   576

Consensus             GGNVLTXEICLGESSXIEXEYXDXKTLLZWAGGGGGGGGAXYXFKXXNGVFRPLLIAAGGGGKAYLEDPXXSLDQAHLEQFENSTAVPGVNGRSGAAGGGGGWNDXTLLPRAGKSLLEGA   712
A. mexicanum melanoid ......R.L......IT.L..Y..N....Q...........S.I..LD.......................KG.SE.....E..K.I.A......A...........S.....S....M...   697
A. mexicanum wildtype ......R.L......IT.L..YEN....Q...........S.I..LD.......................KG.SE.....EL.K.I.......A...........S.....S....M...   697
D. rerio              .R.PQ.HK.......V..DGFDSDGSA.K...........T.IYRME..QPL.....................ES.Q..SFR..Y..D.T.S..S...........S.VSS.SW.....V..G   690
G. gallus             .A.PQ.QK.......V..ED.KKK.D.KD...........T.V.RQKD.I.E...............KAQDN...DVP..........S..T.........Q.V....Q........G   694
G. seraphini          .....MKQ.......V..E..K.KRA..E...........T.V..QE....K..I........S.M.H.DS...HMY......N.........I..Q.......K.I.HF.S.........   658
X. tropicalis         .K.E..K...M.K..I..DN.D.QNRFSE...........S.V..EV..E.I...V............QEDP..FI.P.R...N.....P..M............I.E..V.....F..   696


Consensus             EGGQXCPQAXAKLQWXTSGGFGGGGGACTSGGGGGGYRGGNASEXDDITXDGQDGISFVXPXGXIFLQPLAAMESHGEVQIZVFLNCSHCVSXNCKRDPDTKLVVCJCXSGEVLASDNVT   832
A. mexicanum melanoid ...KV....S.N.H.T......................K.......F..L.T..E.....MS.A.E.................E.....N....G......R....T.I.K...L.....IN   817
A. mexicanum wildtype ...KV....S.N.H.T......................K..H....F..L.T..E.....MS.A.E.................E.....N....G......R....T.I.K...L.....IN   817
D. rerio              Q..SS..E.LSV.G.A.F..........SA..........D.PLL...SA.....L...H.M.K...............AE.V.Y......KTQS....E.....IL.L.D.D....P....   810
G. gallus             ....A.H..L.....A........................H..KN....AG..Y.....N.I.E...H....V.ND...EVQIY......H.D.........N.P..Q.EM.A...N....   814
G. seraphini          ....S.N.SFS....V.F.........P..A..............RI...IAN....V...N.I.K...V.......D.....QL.......S.RK....Q.L..A..V.LQ.T.....G..   778
X. tropicalis         ....T.YK.IT....H..........P...........Q.......L...FT...Y.V..FH.SVK...H.............KL.V.....YTD......INRS....L.EV.T......T.   816

Consensus             CTX-----------------------------PXXEXHXPLPLILAVVXATVILGVIXTCGSLSXVYHRKKQQLEGARIRLQSSEYKLSKIRTSTIMTDYNPNYCFAGKAATLSELKEI   922
A. mexicanum melanoid ..E-----------------------------.DL.E.F.I....S..T........F...G..V...................................................   907
A. mexicanum wildtype ..E-----------------------------.DL.E.F.I....S..T........F...G..V...................................................   907
D. rerio              .AGTKHSLCQFINKHLQHNSSPLVCPPLVVPMGSLADGPPS.VF.M..IVS..VT..VL..A..TLI.Y...NH.HAV......P.........S...........GYF....S.....V   930
G. gallus             ..----------V-----------P------QGHIP.G.L....L....AVI.M..M.L.G....II..L..........A....P.........A.....................   907
G. seraphini          .RG--------T------------------------.RPRH........I.A.V..IVL..S..AI..R...P..V..QA..................F.............V...S......L   866
X. tropicalis         .VV-----------P-----------K-----W.LA.KQL..S.....LI.....I.FA....F.....K.....V....T........S.I.................G.S.....V   909

Consensus             PRKNISLLRALGHGAFGEVYEGTVVGXAGDPNPLQVAIKTLPEIXSEQDEMDFLMEALIISKFSHQNIVRCIGVSLQTLPRFILLELMAGGDMKSFLRQNRPRVNQPSSLTMLDLLNMAR  1042
A. mexicanum melanoid ...........................M.................S...........................V..................................LK.............  1027
A. mexicanum wildtype ...........................M.................S...........................V..................................LK.............  1027
D. rerio              .....T................Q.L.MN.ENTAM..........C..............M.................I..........T.........L....T.HS...S..E..H...  1050
G. gallus             ...........................I................VC...................N....Q.......................................V...Q....I..  1027
G. seraphini          ..R....I...............T.I.E.LT..R.........SC.........................Q......M.....................EK...T.I.Q.......  986
X. tropicalis         .....T................M...IS.................C...........................................S............WI....T.S.........  1029

Consensus             DIACGCKYLEENHFIHRDIAARNCLLTCXGQGRVAKIGDFGMARDIYRASYYRKGGRAMLPVKWMPPEAFLEGIFTSKTDTWSFGVLLWEIFSLGYMPYPCKTNQEVLEFVTSGGRMDPP  1162
A. mexicanum melanoid .....S....D.................G.........................................................................................  1147
A. mexicanum wildtype .....S....D.................G.........................................................................................  1147
D. rerio              ...L..R......................P.PD...........................................C...................................G.......  1170
G. gallus             .............................T.A..I...................................................................................  1147
G. seraphini          .............................S.KD..........................................V..........................................  1106
X. tropicalis         ...........................YS.T............................................V..........................................  1149

Consensus             KNCPGPVYRIMTQCWQHSPEYRPBFXTILERIKYCTQDPDVINTELPVECXPAPEXEGSTXMRPAGSSGMTPLLXSPSLXXQLXXR----SXEAQRXGPKL-VXCPQELLVESLGGWXVX  1277
A. mexicanum melanoid ........................D.T...............V........S....E...AG............VM.....TQ..VQ.----.P.....L...I-.E......I.D.....A.Q  1262
A. mexicanum wildtype ........................D.T...............V........S....E...AG............VM.....AQ..VQ.----.P.....L...I-.E......I.D.....A.Q  1262
D. rerio              .S..............C..H..N.T......N............P.....G.PV.E..G.VI..D..GS.....VAR..SQDASP.ASIT.VTP.ALK.R.QLQR.VH.TQ.VGTYRETL  1290
G. gallus             ........................N.S...................Q..M..S.T..D..N.VV..SVP..IV...V.....TP.MTPK----.LDTHCT.H..-.Q.........N.SNRTAR  1262
G. seraphini          .K................C..D.S.......................I..G..S.D..ESI.Q.TA.E-IV...AT.I.RPK.TSG----KL..EHTVH..-GDH..........D.NMK  1220
X. tropicalis         .................T..H..N.S......S...........P....SG....D....SI..NSCN.LN.IMS.QPIAPL.---------DT.STEQ..-GDV.......N.A..NIK  1259

Consensus             EPSLP---CLSDSTSAXWFQ-QNSSSIAESXLQT-XXRLKNKXKNLWNPTYGSWVME-------------S--X-CRSSXNXNPKXTVEQEDS--GFDGNCSSSPSSR-----------A  1363
A. mexicanum melanoid .....---........P...-.........CT...-AQ..T..N..........L..D-------------.--A-....Q.T...P.......--.............-----------.  1348
A. mexicanum wildtype .....---........P...-.........GTM..-AQ..T..N..........L..D-------------.--A-....Q.T...P.......--.............-----------.  1348
D. rerio              ..CWAEPVPA.GVCPGP.L.VPEHRPCSR..SSSSGSQK....T...........L.SFGRGKSALCHTQ.MPLS.NPTSVSA.SS.S.HT.PVVEVNA.V.A..PPSAAPSQTTLTPT.  1410
G. gallus             G....---....FN..S.I.--..NQKL.FSNPP-TH.....T............L.------------N.----.HL.PISKL.V.P.R...--.............ASP--------.  1350
```

```
G. seraphini            S...S---..T..N.KS.IH-......EQ.K..I-MH.....PQ.............-------------G------IGS.CSF.VVLD....--........C....-----------.  1303
X. tropicalis           T.G.Q---TVAE..EGS...-S.L.TTT.PK...-THK....S..............STC------------------------------------------------------------  1374

Consensus               SPXRGQ---CAHLDIGGI-ELVKLQNFPCGNVNYAYDDLCFEXDG------------XSXVDTRDRXAVLRSXSLCGXGLVLR-------------KXEKPXGERDSGLSLSEDLSXTPV  1454
A. mexicanum melanoid   ..N...---.........-.......................M..------------A.A......T.....A....E.....-------------.QD..P..............L...  1439
A. mexicanum wildtype   ..N...---.........-.......................M..------------A.A......T.....A....E.....-------------.QD..P..............L...  1439
D. rerio                A.S.KSPTGA.GVSLATVMD.A...S...........EQSY.TESLPVVVSKSLEPST.SAA.SSLV.LSQPG.FTHKP..K.HASYGHEDVRRYTQP...TRD....F.......V...  1530
G. gallus               ..S.NN---.P.......-......................GT.H-S-----------PS.L.GV.A.....S..HTDEKPFY-------------.T..AAR.........D...V...  1441
G. seraphini            ..DQDH---GP....N.L-.VD...T..S..........NY.I.N-T----------------K..A....NGRFQVDAQI..-------------.P..IA..........D.FNI...  1389
X. tropicalis           ------------------------------------------------------------------------------------------------------------------------  1314
```
